# Tobacco industry messaging around harm: Narrative framing in PMI and BAT press releases and annual reports 2011 to 2021

**DOI:** 10.3389/fpubh.2022.958354

**Published:** 2022-10-18

**Authors:** Iona Fitzpatrick, Sarah Dance, Karin Silver, Marzia Violini, Thomas R. Hird

**Affiliations:** Tobacco Control Research Group (Partner in Stopping Tobacco Organisations and Products), Department for Health, University of Bath, Bath, United Kingdom

**Keywords:** tobacco, harm reduction, policy frames, thematic analysis, commercial determinants of health

## Abstract

Influencing public perception is a key way in which all transnational corporations (TNCs) maintain market dominance and political power. Transnational tobacco companies (TTCs) have a long history of leveraging narratives to serve commercial ambitions. The global reach of these companies' narratives has been highlighted as a challenge in combatting public health problems caused by tobacco. The corporate power of TTCs is carefully curated, and their narratives play an important role in the setting of governance dynamics at local, national and transnational levels. This qualitative work explores and compares the language used by British American Tobacco (BAT) and Philip Morris International (PMI) around harm, harm reduction and terms used to refer to newer nicotine and tobacco products, including electronic cigarettes and heated tobacco products. We systematically examine framings used by these two TTCs through company reports published between 2011 and 2021. Qualitative coding was carried out by four coders, according to a protocol developed specifically for this work. We firstly identified the presence of pre-selected keywords and then assigned chunks of text containing those key words to one or more associated frames drawn from Boydstun's policy frames codebook (2013). Qualitative coding identified the most common frames from Boydstun's codebook and thematic analysis highlighted three overarching themes. The most common frames assigned were “capacity and resources”, “health and safety” and “economic” frames. The overarching themes were individualization, normalization, and regulation. These themes capture how both BAT and PMI use particular framings to downplay the role of TTCs in the perpetuation of population- and individual-level harms related to tobacco use. They seek to normalize their role in public discussions of health policy, to cast themselves as instrumental in the redress of tobacco-related inequalities and shift responsibility for the continuation of tobacco-product use onto individual consumers. These tactics are problematic for the effective and impartial development and implementation of local, national and international tobacco control agendas.

## Introduction

Corporate storytelling is recognized as an important tool used by transnational corporations (TNCs) to explain corporate decision making to the public, determine perceived limits of organizational responsibility and improve reputation, both internally and externally (1–4). As well as directly impacting public perceptions of the corporation, the narratives of TNCs, including transnational tobacco companies' (TTCs), permit these institutions to influence health governance agendas at local, national and international levels (5–7). The power of TTCs in shaping public health has been well-documented (4, 8), and recent research suggests that their public facing narratives work to obscure schemes of profit maximization (9) and ensure the long-term survival of the business (10). As such, these narratives pose a threat to the reduction of social and economic costs of tobacco-related diseases and to the successful implementation of tobacco control policies at a global scale (11, 12).

The link between smoking and disease, including lung cancer, was apparent to clinicians and the tobacco industry as early as the 1930s and was well-evidenced by the 1950s (13). TTCs attempted to limit reputational damage, maintain profitability and counter threats to cigarette sales by introducing misleading product-based terms that implied reduced harm, such as “light,” “ultralight” and “low tar” (14, 15) and through the co-option of harm-reduction messaging (16).

Existing evidence suggests newer products are an important vector in the expansion of harm-reduction messaging of TTCs. This messaging includes discussions on the role of both traditional oral tobacco, including menthol products (17), and newer nicotine products such as electronic nicotine delivery systems (ENDS) including e-cigarettes; heated tobacco products and snus-style nicotine pouches (18–20) in reducing tobacco-related harms, and has been developed over a number of years (10). Public understanding of associated harms is complicated by the diversification of the tobacco-product landscape (21); which challenges “traditional conceptualizations of smoking and non-smoking” (22), and normalizes tobacco use. Recent evidence suggests that tobacco industry language, specifically language relating to heated tobacco products and ENDS, continues to “foster confusion” among consumers (23) and influences the beliefs of both tobacco users and the public about tobacco-related harms (24–28). The persistence of misleading product-related language is most likely a manifestation of the long term strategy of normalization, which seeks to rebuild credibility to boost sales and profits (10). Denormalization is threatening to TTCs as it can be effective in reducing smoking initiation and increasing intention to quit (29). Philip Morris International (PMI) and British American Tobacco (BAT) have used similar tactics to downplay tobacco-related harms; leaked documents highlight normalization as a strategic focus for PMI (30), while BAT have used advertising to misleadingly emphasize nicotine's apparent similarity to other popular commercial products including chocolate and coffee (31).

Narratives are critical to a number of tactics used by TNCs, including the manipulation of media (32, 33) as well as messaging in advertising and product promotion (23). Understanding TTC narratives, including their framing, could facilitate the development of effective monitoring capabilities and the countering of TTC influence over public health policy and the regulation of advertising and promotion of tobacco products.

This research draws on previous models of TTC interference (8, 32, 34) and focuses on the evaluation of public messaging used by BAT and PMI relating to “harm” and “risk” (16, 35–37). We use a framework not previously used in the analysis of TTCs (Boydstun et al.). In doing so, we show which narrative frames are most commonly used in tandem with newer product terms and the development of harm-related language over time for both BAT and PMI.

## Materials and methods

### Data collection

PMI and BAT were selected based on their market value [largest and second largest TNCs worldwide in 2021 (38)] and existing evidence of their efforts to normalize tobacco product use and downplay tobacco product related harms (16, 30, 31). While PMI and BAT have developed different product portfolios across their global markets, both have used the language of harm and risk in their corporate and promotional material. We collected all annual reports and a sample of press releases of both PMI and BAT published between 2011 and 2019 (inclusive).

#### Keywords

All annual reports were saved. Press releases were filtered for inclusion based on the use of any of researcher-identified key words relating to harm reduction, these were: “harm^*^,” “risk^*^,” “HTP^*^,” “heat^*^,” “e-cigarette^*^” and “vap^*^.” We did not include the collective term “next generation product” (NGP) which has been used by both companies in the past, as this has been superseded by specific product terms. Both annual reports and press releases were downloaded from the company webpages and subsequently saved in NVivo, which was used for qualitative analysis.

Press releases and annual reports covering the relevant date range (2011–2020) containing any of the pre-identified keywords were downloaded directly from company webpages (www.BAT.com and www.PMI.com). Results returned in the search for press releases are shown in Table 1.

**Table 1:** Press releases returned for each target keyword.

**Target keyword**	**Press Release results returned**		**Total**
	**PMI**	**BAT**	
Harm*	76	7	83
Risk*	103	13	116
HTP*	0	0	0
Heat*	39	13	52
e-cigarette*	13	8	21
Vap*	21	20	41
**Total**	252	61	313

The PMI Press releases cover the date range 16/11/2011–25/9/2020, and the BAT press releases 23/2/2011–25/1/2021. Many of the press releases contain more than one of the filtering keywords. Duplicated press releases were deleted prior to the start of analysis. Table 2 summarizes the number of results returned for each pre-identified keyword across press releases and annual reports by year.

**Table 2:** Results returned for each keyword, split by year and sample type.

**Keyword**	**PMI annual reports (individual mentions)**										
	**2011**	**2012**	**2013**	**2014**	**2015**	**2016**	**2017**	**2018**	**2019**	**2020**	**2021**
Harm*	5	8	11	26	24	39	43	38	43	–	–
Risk*	55	62	77	114	122	116	118	147	132	–	–
HTP*	0	0	0	0	0	0	0	0	0	–	–
Heat*	0	1	8	16	14	24	130	165	140	–	–
e-cigarette*	0	1	13	4	5	1	3	11	13	–	–
Vap*	0	0	1	23	29	28	17	28	37	–	–
	**BAT annual reports (individual mentions)**										
Harm*	21	18	25	16	13	16	16	21	26	–	–
Risk*	335	255	226	244	259	261	338	426	446	–	–
HTP*	0	0	0	0	0	0	0	0	0	–	–
Heat*	3	3	5	13	13	25	22	26	23	–	–
e-cigarette*	0	7	7	16	17	10	22	20	13	–	–
Vap*	0	0	0	0	30	35	68	114	164	–	–
	**PMI press releases (count)**										
	2011	2012	2013	2014	2015	2016	2017	2018	2019	2020	2021
Harm*	1	2	5	7	0	15	26	14	9	6	–
Risk*	4	5	14	6	1	15	26	10	23	6	–
HTP*	0	0	0	0	0	1	4	1	0	0	–
Heat*	0	0	0	0	0	4	10	10	19	7	–
e-cigarette*	0	0	3	2	1	1	1	3	2	1	–
Vap*	0	0	0	1	0	2	1	2	17	6	–
	**BAT press releases (count)**										
Harm*	1	1	1	1	0	2	3	4	1	3	0
Risk*	1	1	0	1	0	1	6	6	4	6	1
HTP*	0	0	0	0	0	0	1	0	1	1	0
Heat*	0	0	0	1	2	2	6	5	4	5	0
e-cigarette*	0	1	1	1	2	2	0	3	2	3	0
Vap*	0	0	0	0	3	4	6	5	7	3	0

NVivo was used for sample storage, qualitative coding, and data analysis.

### Assigning frames

Boydstun et al.'s Policy Frames Codebook (39) was used as a framework to code paragraphs that contained any of the target keywords. Relevant text was coded in paragraphs and undertaken by four coders. All relevant text was coded by IF and double coded by SD, MV and KS.

Every paragraph containing any of the keywords was coded firstly to a target keyword and secondly to one or more of the frames from the Boydstun framework. Where two concurrent paragraphs contained either the same keyword or where coded to the same frame, they were coded as a single block for timeliness. It was agreed at the outset that “other frames” would be used sparingly and ideally only after discussion with another coder. All content coded under “other frames” was discussed amongst the coding team.

### Thematic analysis

Summary coding data and memo notes made by each coder (either in NVivo or by hand) during analysis were used as a basis for group discussion between coders once all coding was completed. No upper or lower limits on overarching themes were agreed prior to the identification of themes.

A stepped process of thematic analysis was used to identify overarching themes, through the close examination of data. Repeated discussions with the full coding team enabled the development of a broad consensus on key concepts, frames and findings (40).

In addition to thematic coding, we used the freeware corpus analysis toolkit AntConc (41) to generate word counts for annual reports and press releases for both BAT and PMI, as well as the total word count of the sample. AntConc was also used for keyword querying and the building of our glossary. AntConc was chosen over NVivo for this due to “Keyword In Context” (KWIC) result readability.

## Results

The relationship between key terms, individual codes and overarching themes is reported narratively. Specific examples are used to illustrate patterns or anomalies in the data.

### Summary

The total volume of text analyzed was 1,872,983; the annual reports ran to 1,768,053 words, and the press releases to 104, 930. We used keyword in context (KWIC) searches performed in AntConc to develop a glossary of related terms and phrases.

### Glossary of related terms

Harm^*^: Harm, Harmful, Harms, Hamonization, Harmonising, Harmony, Harmonizing, Harman, Harmonise, Harming, harmonises.

Risk^*^: Risk, Risks, Risky, Risked.

HTP^*^: THP.

Heat^*^: Heated, Heating, Heat, Heatsticks, Heats, Heatstick, Heatcontroltm, Heatcontrol, Heath, Wheaton.

e-cig or e-cigarette^*^: e-cigarettes, electronic cigarettes, e-cigars.

Vap^*^: Vapour, vapor, vaping, vapewild, vape, vaporising, vapeexplained, vapers, vapors, vaporizations, vapourisers.

Identified as not relevant to analysis: harmonization, harmonising, harmony, harmonizing, harman, harmonise, harmonises, heath, wheaton. Noted as of interest but not included as codes: e-vapour, e-vapor, e-liquid, e-liquids, e-hookah, e-lites, modified cigarettes, vape pens, advanced refillable personal vapourisers, electronic pipes.

In addition to the glossary of related terms (above), we also compiled a glossary of related phrases, for both “harm” and “risk.” Phrases were classified as being between 2 and 6 words long.

### Glossary of related phrases

Harm^*^: Harm reduction, tobacco harm reduction, population harm, harm to adult smokers, reduction of harm to the population, (the) harm reduction principles, reduce the harm, risk of harm, the principle of harm reduction, harm economic, social and political development, serious harm, public health goal of harm reduction, public health objective of harm reduction, harmful and potentially harmful constituents, harmful and addictive, potentially less harmful, less harmful than other tobacco products, less harmful than regular cigarettes, harmful to health, harmful components, the harms of smoking, serious harm, less harmful products.

Risk^*^: risk management, reduced-risk products, risk of harm, not risk-free, individual risk, market risks, less risk of harm, “risks, uncertainties and inaccurate assumptions,” risks and rewards, refinancing risk, principle risk factors, key risk factors, system of risk, issue of risk, risk factors, financial risk, risk of smoking-related diseases, assumption of the risk, risk continuum, continuum of risk, risks of smoking, less risky alternatives, risk of loss, risk registers, exchange risk, translation risk, reduction of risk for the individual, credit risk, reduced disease risk, risk spectrum.

### Keywords

#### Harm^*^ and risk^*^

**Figure 1:**
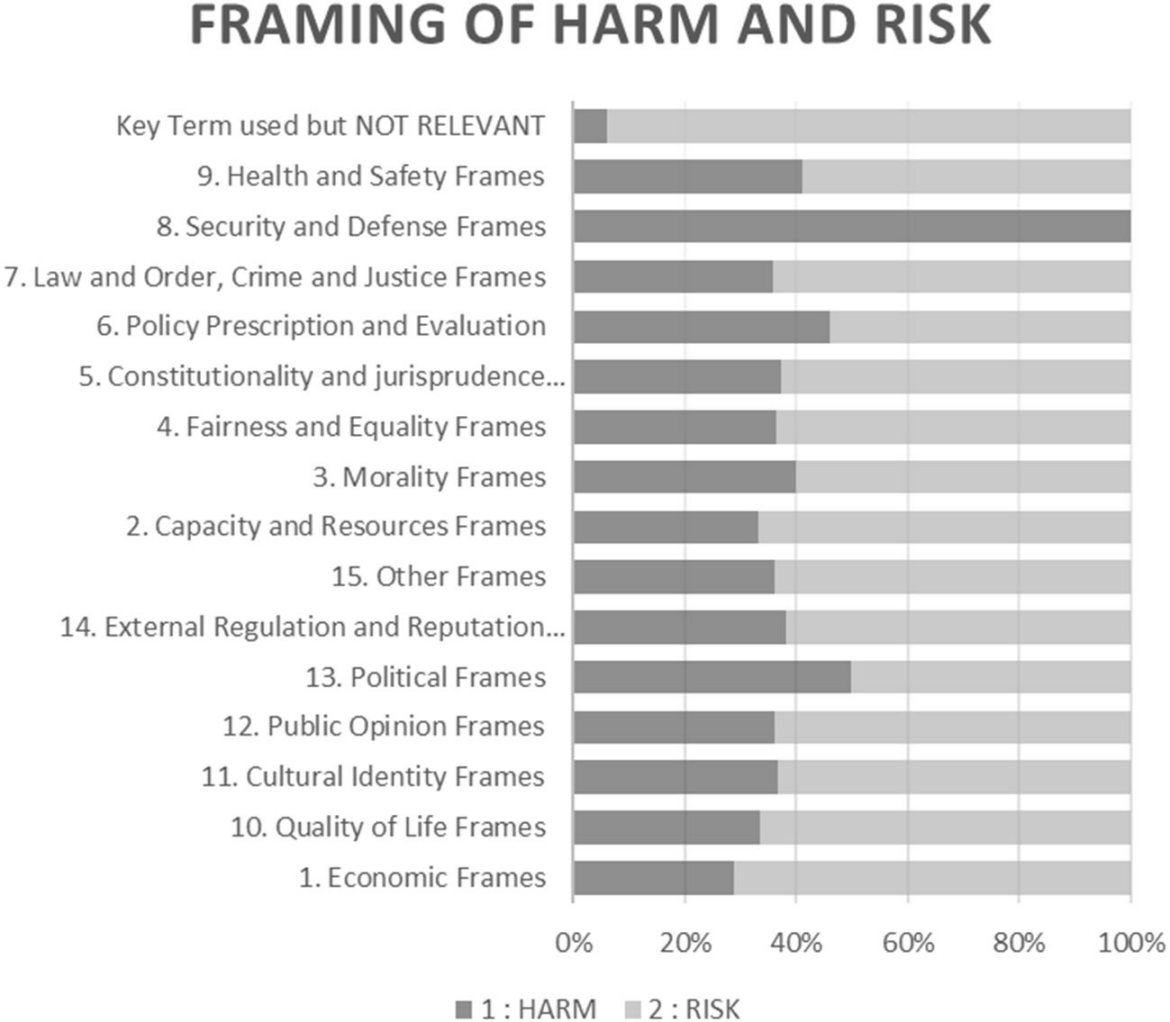
Split of content between harm and risk across policy frames.

As Figure 1 shows, content containing the keyword “risk” or “harm” was spread across frames. ‘security and defense' was the only frame to show exclusive preference to “harm” over “risk.”

Content containing the word “risk” was overwhelmingly coded as “not relevant” to our analysis. These framings concerned the conceptualization of risk not directly related to newer nicotine and tobacco products and not containing any of the other pre-identified keywords. Content deemed not relevant coded under “risk” included, but was not limited to, topics such as security risks, liquidity risks, market risk, digital risk, risk management, risk registers, inflations risks and reputational risk.

#### Newer product terms (HTP^*^, heat^*^, e-cigarette^*^, vap^*^)

**Figure 2:**
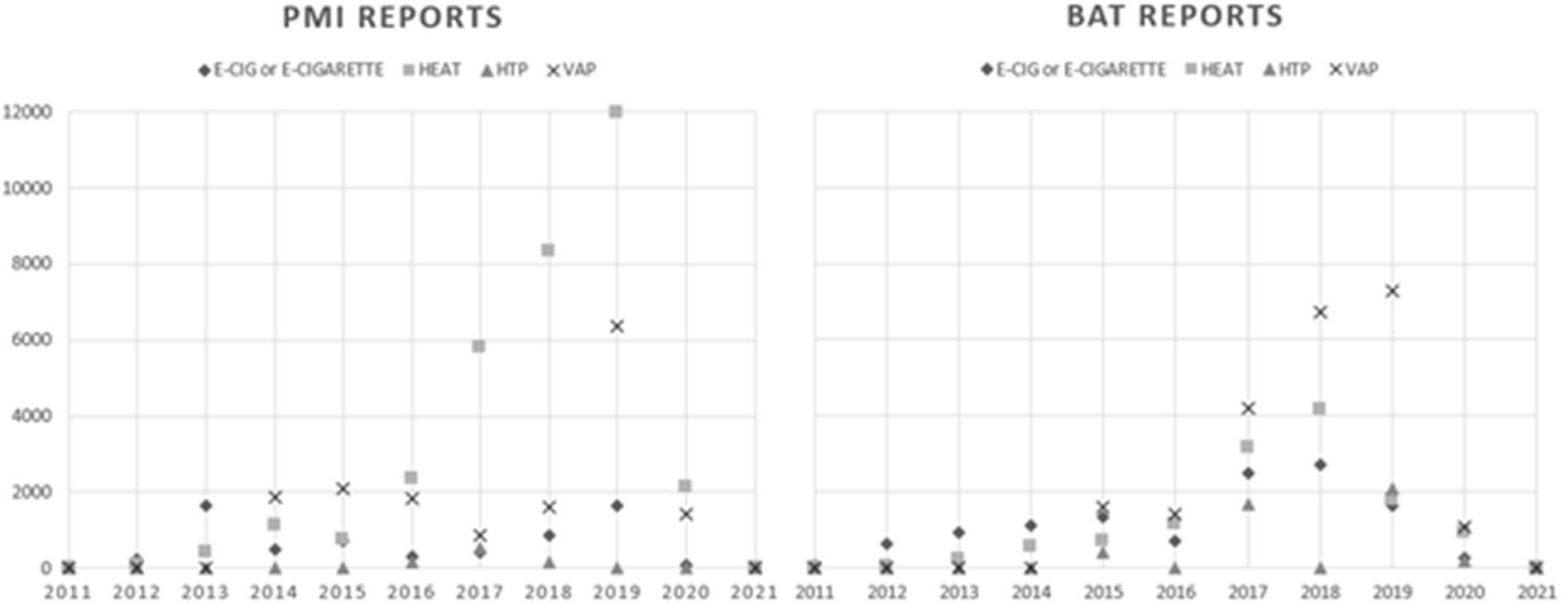
Frequency of newer nicotine and tobacco product terms.

All the keywords HTP (27.6%), heat (23.09%), e-cigarette, vap and their variants were most commonly coded to “capacity and resources” frames. 27.6, 23.09, 19.95, and 21.57%, respectively. “Economic” framings were the second most coded frame to all keyword content. We noted an increase in the frequency of all product-related key words over time in the reports of both BAT and PMI. “heat” appears in the annual reports in 2012 for PMI but is not mentioned in the sampled press releases until 2016. The phrase “heat-not-burn” is present in every PMI annual report from 2013 onwards. BAT used the phrase in their 2013 annual report, but it does not appear again in the sample. BAT used the phrase “tobacco heating product” (THP) from 2014 onwards, referring to *glo iFuse*. THPs are contextualized as part of BAT's commitment to research and development of “potentially less risky alternatives to smoking.” In BAT's messaging, newer products are “potentially less risky,” whilst in PMI's messaging these heat-based newer products are part of their claimed ambition to “replace cigarettes with less-harmful alternatives” (PMI AR 2017). The language concerning the impact of newer products on health vectors has evolved over time. In 2012, PMI describe their “heating and other innovative systems for aerosol generation” as “the most promising path to reduce risk”; later they begin using the phrase “smoke-free products” that were touted as being “a much better choice than cigarettes” (PMI AR 2017). Neither BAT nor PMI used e-cig/arette^*^ frequently in their messaging. PMI use e-cigarette in tandem with “e-vapor products,” presenting their product *IQOS MESH* as an “alternative for e-cigarette users” (PMI AR 2019). In the earlier years of the sample, BAT frame e-cigarettes as a way to “offer smokers a less risky alternative to cigarettes” (BAT AR 2012), the message varied slightly in their later reports, which describe e-cigarettes as part of their efforts in “offering adult consumers a range of satisfying and enjoyable products with reduced-risk potential in comparison to cigarettes” (BAT PR 28 Nov 2020). Figure 2 shows frequency of product related terms over time for each company.

There was an increase in the frequency in the use of newer product (based) terms between 2017 and 2019 for both companies. Neither company used any form of “vap^*^” before 2013. PMI were first to use any variant of “vap^*^”; “vaporizing” was used in their 2013 annual report, in reference to e-cigarettes. They use several forms of “vap^*^” (Vapor, vaporizing, vaporization, e-vapor” throughout subsequent reports. For BAT, the first use of “vap^*^” was not until 2015. The 2015 BAT annual report, similar to PMI's 2013 report, uses “vapor” in relation to e-cigarettes “vapor products (e-cigarettes),” in reference to “next generation products.” BAT first use “vaping” in their 2016 annual report, but by 2020 that term was used repeatedly and the company launched a website (www.vapeexplained.com) that they claimed was intended to help adult consumers “make more informed decisions about vaping” (BAT PR 3 Dec 2020). In contrast, PMI use the term “vaping” only once, ever, in relation to transformation (PMI AR 2018). Similar to BAT, vaping was linked to individual choice arguments; “we want to provide better alternatives to smoking for those who don't quit” (PMI AR 2018).

We noted a difference in the breadth of framing used by each of the TTCs in content coded to both “harm^*^” and “health and safety.” BAT messaging coded to “health and safety” frames was less specifically health related, tending to reference non-specific “health-risks” (BAT AR 2012) and “product and safety standards” (BAT PR 10 Dec 2018), whilst PMI made more frequent mention of specific health vectors such as “smoking-related diseases” (PMI AR 2012) and “biological responses associated with cardiovascular and pulmonary diseases” (PMI PR 14 Mar 2019).

#### Harm reduction and tobacco harm reduction

We analyzed how “harm reduction” was used by the two companies in their reporting, including the context-specific “tobacco harm reduction.” Both phrases were relatively infrequent, in the reports we analyzed. “Harm reduction” was used 117 times by BAT, and 35 times by PMI. “Tobacco harm reduction” was mentioned 44 times and 12 times, respectively. Both “harm reduction” and “tobacco harm reduction” were connected by both PMI and BAT to concepts of research and science, consumer choice, consumer perception, public health strategies and regulation.

### Frames

**Figure 3:**
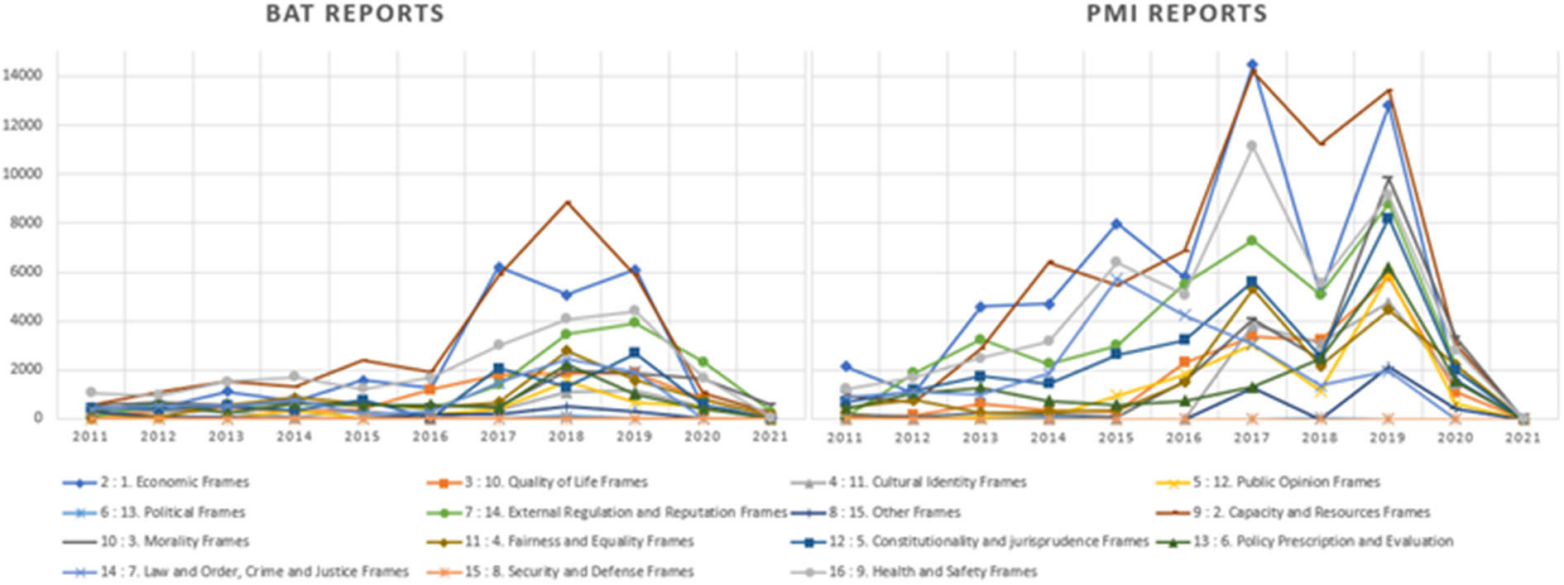
Content coded to policy frames over time in BAT and PMI.

The three most coded frames, in order of individual instances coded were “capacity and resources,” “health and safety” and “economic” frames. Content coded to “capacity and resources” frame was highly variant. It included reference to economic power (market presence, sales, purchasing power of other businesses), expertise, morality and political influence. Example content from the most common frames are shown in Table 3. Figure 3 shows the distribution of content coded to each frame over time, between BAT and PMI.

**Table 3:** Example content from the three most common frames.

**Frame**	**Example**	**Source**	**Observations/notes**
Capacity and resources	Working with external stakeholders on areas of common interest, such as the members of our Biodiversity Partnership.	BAT AR 2011	Political influence
	Through multidisciplinary capabilities in product development, state-of-the-art facilities and scientific substantiation, PMI aims to ensure that its smoke-free products meet adult consumer preferences and rigorous regulatory requirements.	PMI PR 7 May 2019	Expertise
	Since launching its first e-cigarette in the UK in 2013, BAT has made impressive progress, now offering an unrivaled range of innovative and exciting products in more than 40 countries around the world. Over that period BAT has invested over $4bn in the development, manufacture and commercialization of these products.	BAT PR 28 Nov 2019	Economic power
Economic	Our strategy enables our business to deliver growth today, while continuing to invest in our future. Combustible products remain at the core of our business and will continue to provide us with opportunities for growth. However, we also see substantial growth opportunities in the Next Generation Products category and are making significant progress in the commercialization and development of a range of products which offer consumers potentially less risky alternatives to conventional cigarettes.	BAT AR 2016	Economic power
	In the first quarter of 2016, we started the large scale commercial production of heated tobacco units. During 2017, we experienced supply shortages resulting from stronger-than-anticipated demand, primarily in Japan. Currently, we are no longer experiencing capacity limitations. We are integrating the production of our heated tobacco units into a number of our existing manufacturing facilities and progressing with our plans to build manufacturing capacity for our other RRP platforms.	PMI AR 2015	Operational power
	Projects come from public, private, and academic organizations in 18 countries. Grants to be allocated in this first round of PMI IMPACT are approximately USD 28 million.	PMI PR 7 Sep 2017	Economic power
Health and safety	Responsibility is integral to everything we do and is especially important to a business such as ours where our products pose real risks to health. Our determination to act responsibly spans the whole business, from our commitment to addressing the issues of child labor and working with farmers, to looking at how we can help to reduce the harm from our products and lessen our environmental impact	BAT AR 2011	Political, economic, moral
	The meeting was part of the FDA's review of PMI's request to commercialize *IQOS* in the US as a “Modified Risk Tobacco Product”. U.S. law and policy recognize product innovation as important to the 40 million American men and women who smoke. Although the Committee did not agree with some of the specific language of proposed risk and harm consumer communications, it confirmed that the evidence supported the statement that switching completely to *IQOS* significantly reduces exposure to harmful chemicals.	PMI PR 29 Jan 2018	Political
	Harm reduction—Next Generation Products In the world of public health, harm reduction is about developing policies to try to minimize the negative health impacts of a risky activity, without stopping it entirely. For tobacco, harm reduction means offering potentially less risky alternatives to conventional cigarettes to smokers who cannot, or choose not to, give up.	BAT AR 2016	Political, moral

Across content coded to capacity and resources frame, the predominant message was that TTCs were uniquely equipped to deal with several problems, including employment in manufacturing markets, the redress of environmental harms associated with their supply chains and providing “alternatives” for adult consumers who “choose not to give up” smoking. “Science” was related to both “expertise” and “economic power” in ‘capacity and resources' frames.

### Cross-coding of frames

“Economic” and “capacity and resources” coding had some cross over, but “capacity and resources” frames had a broader subject matter than “economic” frames. The “capacity and resources” frame included mention of corporate involvement in and commitment to local and international development initiatives, as well as mention of any staff availability and access to technical and scientific resources. Health and safety frames were more commonly co-coded with “risk” than with “harm.” Content coded to health and safety frames included mention of adult consumers, exposure, “switching,” regulation and multiple mentions of *IQOS* and “next generation products.” Frequent reference was made to legal cases (defendants, courts) in annual reports, whilst mention of “alternatives” to tobacco and cigarettes were noted in both press releases and annual reporting.

### Identification of themes

During data analysis we identified 3 overarching concepts, or themes, that the team felt captured the focus of the harm, risk and product-based narratives of both PMI and BAT: individualization, normalization, and regulation, which we use to structure our discussion below Individualization encompassed arguments made regarding individual responsibility, or any instance where the role of the corporation or any social or political structure was downplayed. Normalization included any reference to the necessary involvement of TTCs in policy processes, mention of sustainability or environmental actions not directly linked to tobacco-product manufacture and associated consumer product development, as well as language that emphasized the perceived polarization of debate or the reported exclusion of the tobacco industry from discussions. Regulation included any mention of internal or external regulatory processes, either specifically product-related or any mention of business or product reporting related to legal processes.

### Coder split and intercoder reliability

Table 4 shows split of content coding performed by each coder according to content type and Table 5 shows split of content coding according to keywords.

**Table 4:** Content coded by each coder across content type (words).

**Coder**	**Annual reports**	**Press releases**	**Total**
IF	153,530	45,800	199,330
KS + MV	131,367	61,031	192,398
SD	88,226	37,036	125,262

**Table 5:** Content coded by each coder across keywords.

	Harm*	Risk*	HTP*/THP*	Heat*	e-cig/arette*	Vap*
Count	576	4,021	162	819	285	772
IF	40,464	142,872	3,464	39,677	16,747	35,713
KS + MV	30,275	111,811	7,931	28,342	15,760	33,887
SD	26,109	90,338	971	28,393	9,287	19,928

We used raw percentage of agreement to measure inter-coder reliability. This allowed comparison of agreement between coders according to the overlap of assigned codes. As the research protocol was paragraph-based, this measure was deemed to be a sufficient indicator of reliability. Secondary coders were treated as a single unit for comparison purposes.

Instances of more than 20% raw disagreement (which included instances where references were coded to a policy frame by only a single coding group) are shown in Table 6. An overlap in coding demonstrates a difference in the assignation of codes between coders, whilst a lack of overlap in coding suggests that one coder did not code the selected text.

**Table 6:** Instances of more than 20% raw disagreement, split by TTC, content type, keywords and frames.

**Attribute**		**Instances of >20% disagreement**	**Mean disagreement (%)**	**Overlap in coding? (Y/N)**
TTC	PMI	632	38.82	Y
	BAT	144	37.02	Y
Content type	Annual report	0	N/A	N/A
	Press release	778	38.48	Y
Keyword	Harm*	12	30.93	Y
	Risk*	17	30.82	Y
	HTP*	8	33.18	N
	Heat*	13	26.48	Y
	e-cigarette*	2	51.06	N
	Vap*	5	24.29	N
Frame	1.Economic	99	48.22	Y
	2.Capacity & resources	60	36.47	Y
	3.Morality	53	42.73	Y
	4.Fairness & equality	53	35.17	Y
	5.Constitutionality & jurisprudence	77	37.21	N
	6.Policy prescription & evaluation	32	44.34	N
	7.Law & order, crime & justice	4	35.41	N
	8.Security and defense	0	N/A	N/A
	9.Health & safety	76	38.04	Y
	10.Quality of life	62	34.57	Y
	11.Cultural identity	47	41.88	N
	12.Public opinion	53	32.55	Y
	13.Political	1	29.37	N
	14.External regulation and reputation	77	38.30	Y
	15.Other	15	47.95	N

There were no instances of coder disagreement in annual reports. Disagreements between sets of coders (IF vs. KS + SD + MV) were more often than not (14/23) due to a coding difference by coders; though in some cases the disagreement was due to missed coding (one set of coders coded content, and the other did not). Difference in coding selection between coders is an expected outcome of qualitative coding, but disagreement due to missed coding highlights inconsistency in the application of the method. The most frequently coded frames (capacity and resources, economic and health and safety) did not correspond to the highest levels of disagreement. Disagreement in coded content was highest in economic and external regulation and reputation frames.

## Discussion

We noted an increase in the frequency of product-based terms over time for both PMI and BAT. Our analysis also highlighted several product terms were used only a few times and in very limited contexts, both findings which support existing research suggesting the tobacco-product landscape is confusing to consumers (23, 26). In addition to product-based language, risk and harm-based terms and phrases, as our glossaries show, were also highly variant. The use of the phrases “harm reduction” and “tobacco harm reduction” was limited, with neither TTC using them consistently. Highly variant language risks both confusion and conflation and might be a barrier to effective implementation of tobacco control policies (21).

TTCs have used the concepts of “harm reduction,” specifically “tobacco harm reduction,” to support their arguments for a role for TTCs and its newer nicotine and tobacco products in addressing the health and environmental burdens caused by traditional combustible products (16). This work shows not only that BAT and PMI continue to make instrumental arguments (based on capacity and resources) for their involvement in tobacco control but that their framings of “harm,” “risk” are often also moralistic and explicitly linked to consumer freedoms. For example, BAT's annual sustainability report includes a section on harm reduction which focusses on it “reducing the health impact” of its business through the development of “New Categories” underpinned by “consumer choice,” “world-class science” and “standards and regulation” (42). TTCs have recently used reduced harm and reduced risk claims in marketing campaigns (43). Whilst it is easy to evidence claims of reduced harm, a potentially more damaging pattern in the language of TTCs is the extension of harm related language beyond tangible environmental and health costs (direct and indirect) into emotional and moralistic arguments, where the responsibility of reducing harm is placed on consumers and their ability or capacity to make “better” choices. Furedi discusses the “inflation of the meaning of harm” (44), and highlights the risk of “concept creep,” where broad definitions can blur the lines between levels and types of harm; which can confuse understandings of harm. The variety of harm-related phrasing in the reports of TTCs could contribute to the perpetuation of a “radically pessimistic” (44) view of the ability of smokers to quit smoking that ultimately serves to enable TTCs to avoid assuming responsibility for the harms caused by their products.

We identified the themes of individualization, normalization, and regulation, as being dominant concepts in the public facing narratives of both companies. Here we discuss each in turn, then highlight the industry's primary imperative of profit making. We end with a note on the risks of confusion and conflation of newer products.

### Individualization

Focus on the rights of the consumer and consumer choice and the connection of these narratives to moral arguments by BAT and PMI is striking. Both companies make claims about doing and being better by serving the interests of marginalized audiences (including smokers) in either the course of their main business or through their philanthropic efforts and contributions to community initiatives.

The emphasis on individual consumer choice in the future-facing narratives of both TTCs allows them to divest responsibility for the harms caused by their products whilst simultaneously positioning themselves as consumer champions. We saw repeated mention of consumer choice arguments offered in support of newer products by both PMI and BAT. For example, BAT claim they are committed to “meeting all of the differing preferences of our consumers, providing them with a choice of outstanding products across the risk continuum” (BAT AR 2016) and PMI argue that their efforts in newer product development will “provide consumers with the assurance that the product information they receive is based on sound science and allows them to make an informed choice based on the risk profile of different products.” Not only does the ‘individualisation' argument covertly blame consumers who ‘fail to choose' the ‘better' products that are presented to them, but it also precludes the TTCs taking responsibility for individual harms caused by their products. Though this argument was played out in this sample in connection to newer products, it is not new; it is simply a reincarnation of already used narratives that framed smoking not as an “addiction” but as a “habit” (45).

### Normalization

The leveraging of corporate social responsibility (CSR) and Environmental, Social and Governance (ESG) reporting can be an advantageous strategy for TNCs, not least TTCs (46). By promoting their goods, image and activities as socially positive or environmentally friendly, TTCs attempt to normalize their corporate action, while diverting public attention from their own damaging practices (47). The adoption of language and frameworks that are well-established and well-respected by audiences beyond tobacco permits TTCs to build influential popular and political support.

Our analysis highlighted several instances in which both BAT and PMI engage in efforts toward normalization through their involvement in CSR and ESG schemes. Both BAT and PMI made mention of harm in relation to global governance schemes, including the United Nations Sustainable Development Goals and other international rubrics like the “Digital Policy Alliance.”

### Regulation

Regulation is an important focus for any TNC specializing in consumer products and is a useful lens through which to examine the tensions between TNCs and systems of power in nation-states. New technologies and the rising influence of TNCs has minimized the power of nation-states (48) and present a considerable challenge for the effective regulation of consumer products that are manufactured, marketed and sold transnationally. This includes the advertising and promotion of tobacco products (49, 50). Regulation can be a powerful mechanism of political and economic control in a neoliberal system. A report from the World Health Organization highlights five ways in which the TI deploy legal arguments in order to minimize or altogether avoid regulation; including leveraging technological developments which could be said to fall outside the scope of existing regulation (this concerns product developments as well as social media marketing and promotion) and the invocation of a continuum of risk that raises the idea of “relative risk” (51).

This work presents a comprehensive view of the TI's use of “capacity and resources” frames, including their funding of technological and scientific research and development in their advancement of harm-reduction related narratives about their business. The dominance of the “capacity and resources” frames, both in terms of expertise in their workforce and in terms of sheer spending power, in discussions of harm, risk and newer products highlights the economic and social power still exerted by TNCs at a global scale. Though many TNCs, TTCs included, participate in voluntary reporting initiatives focused on ESG issues, there are few governance structures in place that ensure or enforce the accountability of TNCs (52, 53), which provides latitude for their messaging to divert consumer and regulatory attention away from the evidenced health impacts of their core business—combustible tobacco products.

### Primary objective: Serving commercial ambition

The survival of the business is of paramount importance to the TNC; TTCs are not unique, they are self-serving and must overcome external pressures in order to survive. The social and economic power of TNCs and TTCs plays out in the detail of the annual reports and press releases we have examined here. PMI and BAT, as two of the largest TTCs, both demonstrate systems of ideas that illustrate inequalities inherent to existing systems of global governance (4, 12), including the centrality of TNCs to the development of regulatory solutions and the emphasis of individual responsibility for the redress of tobacco-related harms. TTCs wield enormous ideational and economic power; their press releases and annual reports show us this.

TNCs play a key part in the creation of problems they seem desperate to solve. TTCs, in particular, have contributed to tobacco-related health burdens that are shouldered in large part by low and middle-income countries (54), where they manufacture many of their consumer products and market combustible products to youth (55). Both BAT and PMI make claims of commitment to the reduction of harm through innovation and investment in newer products in their annual reports and press releases, yet cigarettes remain a large component of their business models (56) and they continue to invest substantial revenues in the development of combustible-focused business (57, 58). TTCs, like other TNCs, use appealing narratives to divert attention from the reality of the social, economic and health damage caused by their business; BAT's “better tomorrow” and PMI's “smoke-free future” are examples of this.

### Newer nicotine and tobacco products and the risks of conflation

The development and promotion of newer products provides renewed opportunity for the manipulation of the public perceptions of tobacco-related harms by the TI. There is a clear historical precedent (13–15) and up-to-date evidence (22–24) already demonstrating the impact of product-based marketing and language on the public perception of harms relating to the use of TI products. This research supplements this body of evidence with an in-depth analysis of the framings of “harm” and “risk” in TI documents over a period of 9 years. The tobacco industry has a history of marketing its products with reduced harm claims which have proved to be false (13, 15, 59, 60). This history of industry misrepresentation is concerning as reduced harm claims are consistently being made newer “next generation,” and “novel” products, including risk-based terms such as “modified risk” or “potentially less harmful.”

Our focus on newer products shows just how central these products are to messaging around harm, risk and harm reduction and highlights how confusing the newer product landscape might be to consumers. A few of newer product descriptors, such as “e-hookah” and “e-cigar” were mentioned rarely and only in very specific contexts—in BAT Annual reports between 2017 and 2019 in reference to the FDA's “Final Rule.” This indicates that some product terms used in regulatory contexts are not yet in common use; a difference in language that may be contributing to the “confusion” around newer products and ultimately impacting the public's ability to fully understand associated risks (23, 24).

The TI's investment in e-cigarette, heated tobacco and oral nicotine products sees them able to re-align themselves with policy-making forums such as the WHO and the UN that have previously been off-limits and to try to rehabilitate their reputation. Harm reduction narratives are being leveraged to collaborate with scientists and circumvent existing tobacco control regulations. Longer-term strategies exert covert power by framing the parameters of debate, reshaping norms and beliefs around the TI and tobacco control, legitimizing TTC positions, and ultimately seeking to make TTCs' agendas appear desirable to policymakers and the tobacco control community. TTCs leverage distracting narratives about transformation in attempts to redefine dominant narratives and shape the language of the debate and the attribution of responsibilities for action linked to tobacco harm reduction, whether this is environmental, social or health related. In the materials we analyzed, harm and risk were portrayed as existing across a “spectrum,” which may be a strategy for the normalization of harm to consumers. Existing research has suggested that harm reduction is a possible alternative to a “zero-tolerance” approach to tobacco control (61), but uncertainty over the effectiveness of harm reduction remains. Health practitioners, academics, advocates, and regulators seem unable to reach a consensus over the best routes forward. A key part of this difficulty is the role of industry in driving particular agendas concerning harm reduction, including the perpetuation of “relative risk” and the insistence on the power of consumers to make “better” choices.

### Strengths and limitations

This research has relied heavily of the subjective interpretation of codes as well as corporate content. However, the use of qualitative coding frameworks is a well-established method and all content included in our analysis was double coded. Coding was discussed between first and second coders to reduce individual bias. The high degree of agreement between the coding of the majority of the frames demonstrates the overall applicability of the coding framework to the selected data sample. However, disagreement between coders was common in press release content, which suggests that the method of chunked coding across many frames may be problematic in the systematic analysis of short-form content. Similarly, the use of raw agreement and disagreement (% of intersection between coders) for one-to-many coding has been criticized for insufficiency as it fails to account for coder agreement resulting from chance (62). The inclusion of “HTP^*^” as a filter missed corporation-specific framings of heated tobacco products, such as tobacco-heating products (THP), which is a term used by BAT. However, the inclusion of “heat^*^” compensated for this. Therefore, we suggest that the key terms used for data sampling should be revised and refined according to the vocabulary of the TNC under study.

## Future work

A recommendation for future work in this vein would be to further examine the use of such frames to explorations of corporate narratives and to consider the use of alternative data samples in answering research questions related to those matters. For example, when examining illicit trade; annual reports and press releases may not be a suitable data source.

Additional work in this area would usefully include an investigation into the impact of the narrative frames we have discussed here, and an examination of how such narratives influence social norms. Future research could also draw out the similarities and differences in relation to varying product portfolios in different geographies, the regulatory landscape and specific instances of policy interference.

## Data availability statement

The datasets presented in this article are not readily available due to copyrighted content. The source data is not owned by the authors and coding data cannot be uncoupled from the source data. Requests to access the datasets should be directed to the University of Bath Research Data Archive: https://doi.org/10.15125/BATH-01196.

## Author contributions

IF and TH conceptualized the research. IF, SD, KS, and MV were involved in the selection of relevant scientific literature. IF developed the method. IF, SD, KS, and MV analyzed the data and wrote the manuscript with critical inputs and appraisal from TH. All authors have read, reviewed, and approved the manuscript.

## Funding

This work was funded by Bloomberg Philanthropies' Stopping Tobacco Organizations and Products (www.bloomberg.org). The funders had no role in study design, data collection, data analysis, decision to publish, or preparation of the manuscript.

## Conflict of interest

The authors declare that the research was conducted in the absence of any commercial or financial relationships that could be construed as a potential conflict of interest.

## Publisher's note

All claims expressed in this article are solely those of the authors and do not necessarily represent those of their affiliated organizations, or those of the publisher, the editors and the reviewers. Any product that may be evaluated in this article, or claim that may be made by its manufacturer, is not guaranteed or endorsed by the publisher.

## Author disclaimer

The opinions expressed are those of the authors' alone.
